# A bibliometric analysis of two decades of aromatherapy research

**DOI:** 10.1186/s13104-016-2371-1

**Published:** 2017-01-18

**Authors:** Malcolm Koo

**Affiliations:** 1 Department of Medical Research, Dalin Tzu Chi Hospital, Buddhist Tzu Chi Medical Foundation, 2 Minsheng Road, Dalin, Chiayi, 62247 Taiwan; 2 Dalla Lana School of Public Health, University of Toronto, Toronto, ON Canada

**Keywords:** Aromatherapy, Bibliometric analysis, Web of science database, Citation analysis, Scientometrics

## Abstract

**Background:**

Quantitative data are lacking on the profile of published research in aromatherapy. The objective of the study was to investigate the profile of original and review articles under the topic aromatherapy using bibliometric analysis.

**Methods:**

Articles on aromatherapy, published between 1995 and 2014, were retrieved from the Science Citation Index-Expanded database from the Web of Science. The records extracted were analyzed for citation characteristics, including the distribution of publication years, languages, countries or regions, journals, articles, and authors using HistCite 12.03.17. VOSviewer v.1.61 was used to construct bibliometric diagrams.

**Results:**

A total of 549 original and review articles, published in 287 different peer-reviewed journals by 1888 authors, were identified. There was a steady increase in the number of published articles from 1995 to 2014. The majority of the articles was written in English (95.8%) and the United States was the leading country in the total number of published articles (n = 107, 19.5%) *Journal of Alternative and Complementary Medicine* published the greatest number of articles on the topic (n = 31, 5.6%). The article that received the greatest number of citations was published in *Complementary Therapies in Medicine.* Visualization analysis based on co-occurrences of words in the title and abstract revealed three clusters of research topics, including essential oil, intervention, and complementary medicine.

**Conclusions:**

This study provided a systematic overview of productivity and visibility of research work in aromatherapy and the findings could be used for organizing and prioritizing future research efforts in aromatherapy research.

## Background

Aromatherapy can be defined as a controlled use of aromatic plant oils for therapeutic or preventive purposes. It can be applied through aerial diffusion, direct inhalation, and topical applications. The use of essential oils for therapeutic and spiritual purposes can be dated back to ancient civilizations, including the Chinese, Indians, Egyptians, Greeks, and Romans. However, the beginning of contemporary aromatherapy is often attributed to the pioneer work of the chemist René-Maurice Gattefossé and doctor Jean Valnet from the early twentieth century in France. It was not until the 1980s that aromatherapy became popular in the United States and began to gain attention for its potential clinical applications. Nevertheless, despite its popular use in both the community and health care settings today [1, 2], there is still a paucity of empirical evidence supporting the efficacy of aromatherapy in many therapeutic claims [3]. For example, systematic reviews indicated there are still limited evidence to support the effectiveness of aromatherapy in controlling stress [4], relieving labor pain [5], controlling hypertension [6], reducing postoperative nausea and vomiting [7], and improving symptoms of dementia [8]. It is clear that more well-designed and implemented large-scale randomized controlled trials are needed to establish the efficacy of aromatherapy in these areas. In the meantime, analyzing the key journals, authors, and exploring the relationship of topical hotspots of aromatherapy research may provide insight into the scope of aromatherapy research and help researchers to establish research priorities.

Bibliometric analysis is a useful methodology for investigating publication patterns based on citation data of academic literature. Bibliometric methods historically have been used to explore relationships among academic journal citations and to provide insight into the dissemination of research findings. A well-known example of citation index is the Science Citation Index established by Eugene Garfield in 1964 [9]. With the wide availability of bibliometric analytical software, there is a rapid proliferation of bibliometric studies on various medical topics in recent years. For example, bibliometric analyses have been utilized to profile the trend of research on various diseases or interventions [10, 11], to quantify a country’s scientific output [12], to gain an insight into the changes in performance over time in a particular area of research [13], to identify highly cited publication [14] and their characteristics [15], and to explore the hot topics of research in a given field [16].

Bibliometric methods have also been applied in complementary medicine research. Vickers used Medline database to determine a number of features of randomized trials in complementary medicine, including the extent to which they are indexed, the journals in which they are published, dates of publication, and the therapies and conditions studied [17]. More recently, Cramer and colleagues reviewed the bibliometric characteristics of randomized controlled trials of yoga [18]. Hung and Ernst assessed the methodological quality of randomized clinical trials of herbal medicine research between 1977 and 2007 [19]. Kim and colleagues analyzed randomized controlled trials on complementary and traditional medicine in the Korean literature using bibliometric analysis [20]. Moreover, Han and Ho evaluated the global trend of acupuncture research based on the Science Citation Index-Expanded database between 1991 and 2009 [21]. The evidence base of clinical studies of Tai Chi for healthcare was evaluated bibliometrically using the PubMed database, the Cochrane Library, and four major Chinese electronic databases [11]. In addition, the statuses of complementary medicine research in China, Taiwan, and Hong Kong were compared bibliometrically based on publications from 2000 to 2009, identified from the PubMed database and the Journal Citation Reports [22]. Nevertheless, to our knowledge, no studies have specifically explored the bibliometric profile of aromatherapy research. Therefore, the present study used bibliometric analysis to study the profile of research articles on aromatherapy published in the past two decades (1995–2014) and to identify topical hotspots in aromatherapy research.

## Methods

The Thomson Reuters Web of Science website was used to identify research articles on the topic of aromatherapy. The Science Citation Index-Expanded database was selected. The search was conducted on July 1, 2015. The publication period was limited to 20 years, 1995–2014. Original and review articles were selected for further analyses. The records extracted were analyzed for citation characteristics, including the distribution of publication years, languages, countries or regions, journals, articles, and authors using HistCite 12.03.17 (HistCite Software LLC) [23].

The observed distribution of the frequency of authors and the number of their publications was fitted with the distribution function depicted by the Lotka’s law using a computer program to obtain the values of the exponent *n* (i.e., the slope of the log–log plot) and the constant *c* (the fraction of authors with only a single publication) (LOTKA version 1.02). The deviation between the observed and the theoretical distribution function was evaluated using the Kolmogorov–Smirnov goodness-of-fit test [24]. In addition, the frequency of journals and the number of the articles that they contained were also evaluated with the computer program.

In addition, VOSviewer v.1.61 for Microsoft Windows (Centre for Science and Technology Studies, Leiden University, The Netherlands) [25] was used to construct bibliometric diagrams for visualization of co-citation of the journals and co-occurrence of the text corpus extracted from the title and the abstract fields of the articles. Co-citation can be defined as any two items (authors) that have been jointly cited by another item (author). Thus, the more co-citations two items received, the more likely that they are related [26]. The fractional counting method was used when constructing the co-citation network. With fractional counting, if a citing article contains *n* references, each citation will count for only 1/*n* of the overall citations.

For the co-occurrence analysis, the text mining functionality of the VOSviewer first identifies the noun phrases in the text corpus based on the Apache OpenNLP toolkit, and then it converts all plural noun phrases into singular ones. The relevance of the resulting noun phrases was determined by comparing the pattern of their co-occurrences. While noun phrases with a low relevance will exhibit a random pattern of co-occurrence with other noun phrases, those with a high relevance will co-occur mainly with a limited set of other noun phrases [27]. In this study, the noun phrases (hereinafter referred to as “terms”) identified by VOSviewer were also manually inspected. Words of similar meaning or abbreviation (for example, “cam” and “complementary”) were merged into its canonical form. Terms that deemed uninformative such as “year”, “change” and publishers’ name were eliminated to improve the clarity of the resulting network.

## Results

A total of 661 publications on aromatherapy, published between 1995 and 2014, were retrieved from the Web of Science. The distribution of article types is shown in Table 1. Of them, 465 (70.4%) were original articles and 84 (12.7%) were review articles. These 549 articles were included in the subsequent analyses.

**Table 1:** Distribution of types of articles on aromatherapy published between 1995 and 2014 (N = 661)

Article type	n	%
Original article	465	70.4
Review	84	12.7
Meeting abstract	40	6.1
Proceedings paper	25	3.8
Article; proceedings paper	15	2.3
Editorial material	15	2.3
Letter	10	1.5
News item	3	0.5
Correction	2	0.3
Book review	1	0.2
Note	1	0.2

Only “Original article” and “review” were included in subsequent analyses

**Fig. 1:**
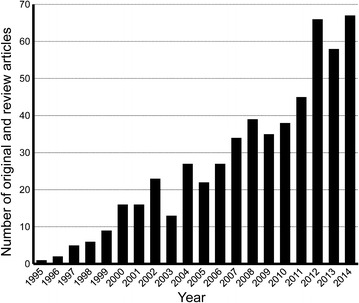
Time trend of the number of original and review articles on aromatherapy published per year from 1995 to 2014

Figure 1 indicates that the number of articles increased steadily during the 20-year period with 67 articles published in 2014. As expected, the majority of the articles were written in English (95.8%) (Table 2). Authors from a total of 58 countries or regions contributed to the 549 published articles. The United States had the most published articles (19.5%), followed by the United Kingdom (17.3%). In terms of citations per paper, Australia was the leading country (34.5%) (Table 3).

**Table 2:** Languages used original and review articles on aromatherapy published between 1995 and 2014 (N = 549)

Language	n	%	TGCS	Citations/article
English	526	95.8	8934	17.0
Korean	7	1.3	22	3.2
German	6	1.1	7	1.2
Japanese	2	0.4	2	1.0
Portuguese	2	0.4	5	2.5
Turkish	2	0.4	3	1.5
French	1	0.2	1	1.0
Italian	1	0.2	0	0
Polish	1	0.2	0	0
Spanish	1	0.2	0	0

*TGCS* total global citation score = total number of citations received

Citations/article = TGCS/number of articles

**Table 3:** Top 15 countries or regions with original and review articles on aromatherapy published between 1995 and 2014 (N = 549)

Country or region	No. of articles	%	TGCS	Citations/article
United States	107	19.5	2024	18.9
United Kingdom	95	17.3	2790	29.4
Japan	57	10.4	781	13.7
Australia	37	6.7	1277	34.5
Germany	29	5.3	409	14.1
South Korea	29	5.3	162	5.6
Brazil	27	4.9	272	10.1
Unknown	24	4.4	257	10.7
Taiwan	18	3.3	86	4.8
Iran	17	3.1	45	2.7
Turkey	17	3.1	34	2.0
Austria	15	2.7	283	18.9
People’s Republic of China	15	2.7	146	9.7
Canada	13	2.4	280	21.6
Italy	12	2.2	170	14.2

*TGCS* total global citation score = total number of citations received

Citations/article = TGCS/number of articles

Of the 1888 authors, Edzard Ernst of the Exeter University, United Kingdom had authored or co-authored the highest number of articles on the topic of aromatherapy (13 articles), followed by Myeong Soo Lee of the Korea Institute of Oriental Medicine, South Korea (12 articles) and Myung-Haeng Hur of the Eulji University, South Korea (10 articles). Moreover, 1654 authors (87.6%) published only one article, 167 authors (8.8%) published two articles, 43 authors (2.3%) published three articles, and only 24 authors (1.3%) published four or more articles on the topic of aromatherapy.

The 549 articles were published in 287 different journals. Table 4 shows the top 20 journals with the highest number of original and review articles on aromatherapy. Six of them were of the Web of Science category “Integrative and Complementary Medicine”. The median impact factor was 1.77 and six were quartile 1 journals, seven were quartile 2 journals, according to the classification of the Journal Citation Reports. Regarding the number of articles published in each journal, *Journal of Alternative and Complementary Medicine* published the most articles (31 articles), followed by *Complementary Therapies in Medicine* (27 articles), and *Evidence*-*based Complementary and Alternative Medicine* (21 articles). In addition, 17 journals contained a range of five to nine articles, eight journals contained four articles, 14 journals contained three articles, 48 journals contained two articles, and 197 journals contained only one article. Furthermore, the top 50 ranking journals (17.4% out of the 287 journals) accounted for 272 or 49.5% of the 549 articles.

**Table 4:** Top 20 journals with the highest number of original and review articles on aromatherapy published between 1995 and 2014 (N = 549)

Rank	Journal	Web of Science subject category	No. of cited articles	%	TGCS	Citations/article	Impact factor^a^ (quartile)
1	Journal of Alternative and Complementary Medicine	Integrative and complementary medicine	31	5.6	315	10.2	1.585 (2)
2	Complementary Therapies in Medicine	Integrative and complementary medicine	27	4.9	1029	38.1	1.545 (2)
3	Evidence-based Complementary and Alternative Medicine	Integrative and complementary medicine	21	3.8	92	4.4	1.880 (2)
4	Phytotherapy Research	Chemistry, medicinal	9	1.6	658	73.1	2.660 (2)
5	Cochrane Database of Systematic Reviews	Medicine, general and internal	8	1.5	118	14.8	6.032 (1)
6	Natural Product Communications	Food science and technology	8	1.5	80	10.0	0.906 (3)
7	Contact Dermatitis	Dermatology	7	1.3	246	35.2	3.747 (1)
8	International Journal of Neuroscience	Neurosciences	7	1.3	296	42.3	1.521 (4)
9	Flavour and Fragrance Journal	Food science and technology	6	1.1	44	7.3	1.970 (2)
10	Journal of Applied Entomology	Entomology	6	1.1	17	2.8	1.650 (2)
11	Journal of Essential Oil Research	Food science and technology	6	1.1	44	7.3	0.787 (3)
12	Palliative Medicine	Medicine, general and internal	6	1.1	252	42.0	2.855 (1)
13	BMC Complementary and Alternative Medicine	Integrative and complementary medicine	5	0.9	25	5.0	2.020 (2)
14	Forschende Komplementarmedizin	Integrative and complementary medicine	5	0.9	22	4.4	1.079 (3)
15	Iranian Red Crescent Medical Journal	Medicine, general and internal	5	0.9	2	0.4	0.634 (4)
16	Journal of Ethnopharmacology	Integrative and complementary medicine	5	0.9	67	13.4	2.998 (1)
17	Journal of Korean Academy of Nursing	Nursing	5	0.9	20	4.0	0.380 (4)
18	Journal of PeriAnesthesia Nursing	Nursing	5	0.9	12	2.4	0.943 (3)
19	Phytomedicine	Chemistry, medicinal	5	0.9	163	32.6	3.126 (1)
20	Supportive Care in Cancer	Rehabilitation	5	0.9	72	14.4	2.364 (1)
	Median				76	10.1	1.765

*TGCS* total global citation score = total number of citations received

Citations/article = TGCS/number of articles

^a^Impact factors were obtained from the 2015 release of Journal Citation Reports Science Edition with 2014 data

Table 5 shows the 10 most cited original and review articles on aromatherapy published between 1995 and 2014. The top-ranking paper, with 357 citations, was published in *Complementary Therapies in Medicine*. The second-ranking paper was a review article published in *Phytotherapy Research* in 2007 with 278 citations. Since the earlier the published year, the longer the duration that an article has an opportunity to be cited, a citation score per year was also calculated to provide a different index for comparison. In addition, these articles were found to rank at the same position based on the number obtained from the Google Scholar citations.

**Table 5:** Ten most-cited original and review articles on aromatherapy published between 1995 and 2014 (N = 549)

Rank	First author (no. of total authors)	Title	Journal (impact factor^a^)	Year of publication	Global citation score	Global citation score per year	No. of Google Scholar citations
1	Thomas KJ (3)	Use and expenditure on complementary medicine in England: a population based survey	Complementary Therapies in Medicine (1.545)	2001	357	25.5	677
2	Edris AE (1)	Pharmaceutical and therapeutic potentials of essential oils and their individual volatile constituents: a review	Phytotherapy Research (2.660)	2007	278	34.8	493
3	Kronenberg F (2)	Complementary and alternative medicine for menopausal symptoms: a review of randomized, controlled trials	Annals of Internal Medicine (17.810)	2002	270	20.8	556
4	MacLennan AH (3)	The escalating cost and prevalence of alternative medicine	Preventive Medicine (3.086)	2002	224	17.2	467
5	Lorenz KA (14)	Evidence for improving palliative care at the end of life: a systematic review	Annals of Internal Medicine (17.810)	2008	214	30.6	418
6	Ernst E (2)	The BBC survey of complementary medicine use in the UK	Complementary Therapies in Medicine (1.545)	2000	210	14.0	460
7	Cavanagh HMA (2)	Biological activities of lavender essential oil	Phytotherapy Research (2.660)	2002	156	12.0	335
8	DeGroot AC (2)	Adverse reactions to fragrances—a clinical review	Contact Dermatitis (3.747)	1997	149	8.3	14
9	Ballard CG (4)	Aromatherapy as a safe and effective treatment for the management of agitation in severe dementia: the results of a double-blind, placebo-controlled trial with Melissa	Journal of Clinical Psychiatry (5.498)	2002	113	8.7	411
10	Ballard CG (7)	Management of agitation and aggression associated with Alzheimer disease	Nature Reviews Neurology (15.358)	2009	108	18.0	181

Global citation score = citation frequency based on the full Web of Science count at the time the data was downloaded

Global citation score per year = global citation score/(2015 – the year of publication)

The number of Google Scholar citations was obtained from https://scholar.google.com/scholar

^a^Impact factors were obtained from the 2015 release of Journal Citation Reports Science Editon with 2014 data

**Fig. 2:**
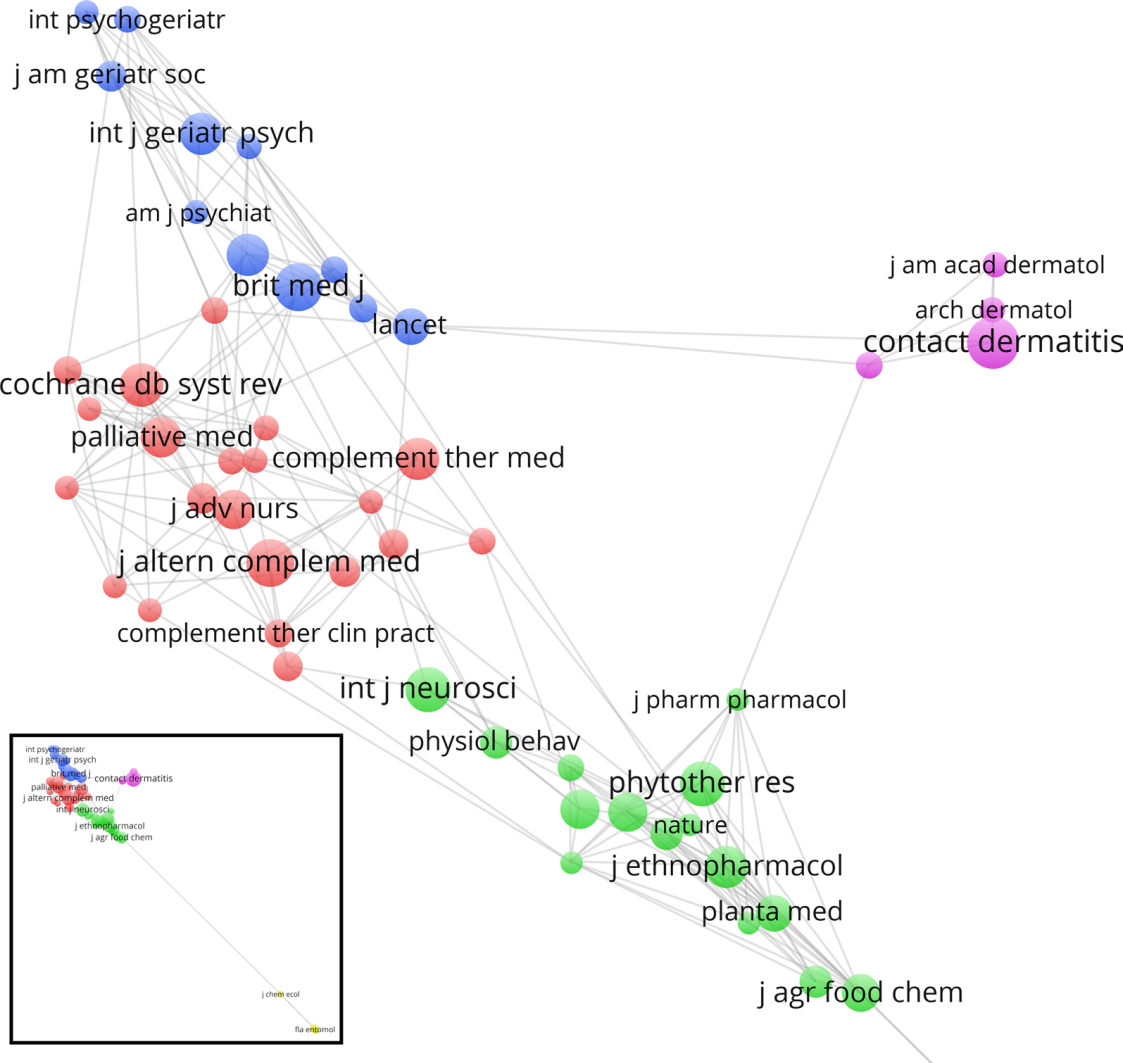
Co-citation network of journals with original and review articles on aromatherapy published between 1995 and 2014 that received at least 50 co-citations. Five clusters were identified: cluster 1 (*blue*, *top left*), cluster 2 (*red*, *middle left*), cluster 3 (*green*, *bottom middle*), cluster 4 (*purple*, *right*), and cluster 5 (*yellow*, *inset*). The inset (*bottom left corner*) shows the full co-citation network map with two journals (*Journal of Chemical Ecology* and *Florida Entomologist*). Clusters located close to each other in the figure indicate related topics

Visualization analysis of the citation data were further explored using VOSviewer. Figure 2 shows the results of co-citation analysis of the 287 journals that received at least 50 co-citations. The size of a circle reflects the number of citations that a journal has received while the distance between two journals indicates the strength of the relatedness between them. Five clusters containing 55 journals were identified. Cluster 1 (red) consisted of 21 journals mainly publishing in complementary medicine and nursing research. Cluster 2 (green) consisted of 15 journals of medicinal chemistry and food science. Cluster 3 (blue) consisted of 11 journals of general medicine and geriatric medicine. Cluster 4 (purple) consisted of four journals focusing on entomology and Cluster 5 (yellow) consisted of four journals in dermatology.

**Fig. 3:**
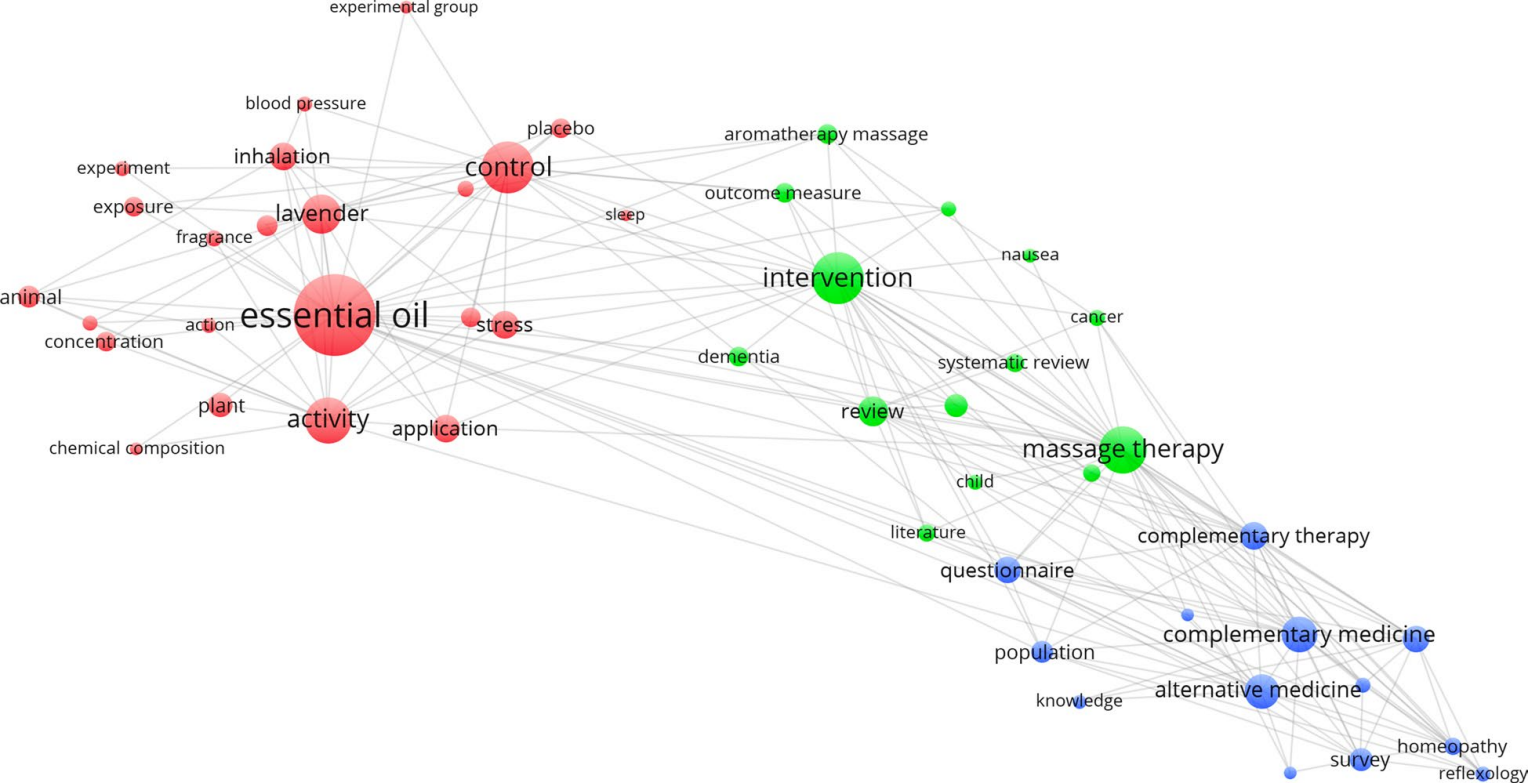
Co-occurrence network of terms occurred in the title or abstract of at least 20 articles on aromatherapy published between 1995 and 2014. Three clusters were identified: cluster 1 (*red*, *left*), cluster 2 (*green*, *middle*), and cluster 3 (*blue*, *right*)

Figure 3 shows the co-occurrence network of terms that occurred in the title or abstract of at least 20 articles. Overall, 84 of the 12,261 terms meet the criteria and the top 60% of the most relevant terms, that is, 50 terms are displayed in the figure. Cluster 1 (red) had 23 terms and the highest co-occurrence term was “essential oil” (216 co-occurrences). Cluster 2 (green) had 14 items with “intervention” receiving 121 co-occurrences followed by “massage therapy” receiving 109 co-occurrences. Cluster 3 (blue) had 13 items with “complementary medicine” and “alternative medicine” receiving 78 and 74 co-occurrences, respectively.

## Discussion

In this bibliometric study, we present the results of publication on the topic of aromatherapy published between 1995 and 2014. The analyses of the growing trend of the number of original and review articles over the period, language used, and countries did not expose unexpected findings. Although over half of the 549 articles originated from authors from non-English speaking regions of the world, 95% of the articles were written in English. This finding reflects not only that English is the de facto global language of scientific communication [28] but also the characteristic of the Science Citation Index, which contains relatively few non-English language journals [29].

Of the 1888 authors, the two most prolific authors in aromatherapy revealed by this study, Edzard Ernst and Myeong Soo Lee were also highly productive in other subfields of complementary medicine. The distribution of the number of articles published by the authors in this study was evaluated with the Lotka’s law of scientific productivity [30]. Based on the statistical distribution of the productivity of authors based on Chemical Abstracts, Lotka observed that “… the number (of authors) making *n* contributions is about 1/*n* ^2^ of those making one; and the proportion all contributors, that make a single contribution, is about 60%.” In other words, the number of authors producing *n* articles is proportional to 1/*n* ^2^ or similarly, the number of journals containing *n* articles is proportional to 1/*n* ^2^. Our study found that only 12 authors contributed to five or more articles while 1654 of the 1888 authors (88%) contributed to only one article. However, the LOTKA computer program [24] failed to fit a Lotka’s power law distribution to our observed data.

On the other hand, the distribution of the 549 aromatherapy articles in the 287 journals fitted well, as indicated by the Kolmogorov–Smirnov goodness-of-fit test, according to the Lotka’s power law. A *n* of 2.424 and a *c* of 0.728 were obtained from the computer program LOTKA and therefore, the Lotka power function can be expressed as Y = 0.728/X^2.424^, where X is the number of articles and Y is the relative frequency of journals with X articles was obtained. According to this formula, 72.8% of the journals can be estimated to contain only one article. The value of *n* is larger than the 2 that originally suggested by Lotka but smaller than the 3.5 that recently reported in a study of citation data from the Scopus database [31]. Previous research indicated that the exponent *n* and the constant *c* could be influenced by the subject area and its productivity, the state of development, the country of origin, the time period of the study, and the length of that period [32].

As anticipated by the power law, most articles were concentrated in several journals. In fact, almost a quarter of the articles were published in only three journals, namely, *Journal of Alternative and Complementary Medicine*, *Complementary Therapies in Medicine*, and *Evidence*-*based Complementary and Alternative Medicine.* Hence, these journals can be considered as the core journals for knowledge dissemination of aromatherapy research.

The top 10 most cited original and review articles on aromatherapy published between 1995 and 2014 were analyzed to reveal the types of research in aromatherapy (Table 5). The top-ranking paper reported the findings from a postal survey of 2669 adults in England regarding their out-of-pocket expenditure on practitioner-provided complementary therapies and over-the-counter remedies [33]. The second-ranking paper was a review article published in 2007. The article described the possible mode of action of essential oils and their volatile constituents and outlined the therapeutic properties of essential oils in aroma and massage therapy [34]. Moreover, half of the 10 most cited articles were review articles. This finding is not surprising since systematic reviews and meta-analysis occupy the highest position in the current proposed hierarchy of evidence [35]. In addition, another possible reason for the high citation counts in review articles is because they are often cited under the introduction section of original articles. A study of the 100 most-cited papers in each of 21 scientific fields during the period 1996–2006 found that a considerable number of the articles in each field were review articles [36].

Visualization analysis was used to create a two-dimensional map of co-citation network of journals that received at least 50 co-citations (Fig. 2). Five clusters containing 55 journals were generated by the VOSviewer. The cluster 1 (red) formed by the 21 journals focusing on complementary medicine were distanced similarly from two other clusters: cluster 2 (green) formed by 15 journals in medicinal chemistry and food science and cluster 3 (blue) formed by 11 journals in general medicine and geriatric medicine. This distribution pattern indicated that while there was high relatedness among the articles within each cluster, moderate relatedness also existed between cluster 1 and 2 and between cluster 1 and 3. On the other hand, cluster 4 (purple) and especially cluster 5 (yellow) had much lower relatedness with the articles in the complementary medicine cluster.

To locate popular research topics on aromatherapy research published between 1995 and 2014, the co-occurrence of terms in the title or abstract of at least 20 articles was analyzed. Three clusters with a total of 50 terms were identified. Cluster 1 (red) consisted of terms related to essential oil such as the mode of administration (inhalation), type of oil (lavender), study design (experiment, placebo), and outcome (stress, blood pressure). The second cluster (green) composed of terms that dealt with interventions (aromatherapy, massage therapy) and medical conditions such as nausea, dementia, and cancer. Systematic reviews and reviews came out as prominent terms because 16% of the articles were reviews. In addition, the appearance of the term “child” reflected a few well-cited survey studies and reviews on complementary medicine use in children [37–40]. Finally, Cluster 3 (blue) contained general terms of complementary medicine, methods of knowledge acquisition (survey, questionnaire), and related complementary therapies (homeopathy, reflexology). The latter co-occurrence could be explained by the fact that these therapies were often included in survey studies [41–43] and disease-specific reviews [44, 45] of complementary medicine along with aromatherapy. The visualization of co-occurrence network could be used not only to show the pattern and hot spots of aromatherapy therapy in the past, but may also help to reveal potential or neglected research areas.

Several intrinsic limitations of this bibliometric analysis should be noted. First, it is possible that some articles could be missed with the use of a single citation database. Further studies can evaluate other databases such as Scopus and Google Scholars and to compare their findings with those from this study. Second, the Science Citation Index database is biased towards English-language journals and therefore, the results should be interpreted as such. Moreover, the non-English language journals included in the Science Citation Index database was found to have a lower impact than those in the English-language journals [29]. Therefore, the comparisons of publication output among countries might be affected.

## Conclusions

This study was the first bibliometric analysis of aromatherapy research. Prolific authors, core journals, and clusters of aromatherapy research in the past two decades were identified. This study provided a systematic overview of productivity and visibility of research work in aromatherapy and the findings could be used for organizing and prioritizing future research efforts in aromatherapy research.
